# Impact of endophyte inoculation on the morphological identity of cultivars of *Lolium perenne* (L) and *Festuca arundinacea (Schreb*.)

**DOI:** 10.1038/s41598-020-64474-7

**Published:** 2020-05-07

**Authors:** J. D. Patterson, F. Lafaillette, S. Wöster, N. Roulund, S. Charrier, T. J. Gilliland

**Affiliations:** 1 Agri-Food & Biosciences Institute, AFBI Hillsborough, Northern Ireland, UK; 2 GEVES, Anjouère Experimental Unit, Domaine de l’Anjouère, La Pouëze, 49370 Erdre en Anjou, France; 3 Bundessortenamt Prüfstelle Scharnhorst, 2, 31535 Neustadt am Rübenberge, Germany; 4 DLF Trifolium, Ny Oestergade 9, 4000 Roskilde, Denmark; 5 Barenbrug Holland BV, Stationsstraat 40, 6515 AB Nijmegen, Netherlands; 6 Institute of Global Food Security, Queens University Belfast, Belfast, UK

**Keywords:** Genetics, Genotype

## Abstract

Grass endophytes have been shown to confer enhanced environmental resilience to symbiont cultivars with reports of modified growth. If inoculating with an endophyte (E+) made an accession morphologically distinct from its registered endophyte free (E−) accession, there could be protection and ownership issues for testing authorities and breeders. This study investigated if, in official Plant Breeders Rights (PBR) field trials, the morphological characteristics of E+and E− accessions of perennial ryegrass and tall fescue cultivars were sufficiently modified to designate them as mutually distinct and also distinct from their definitive accessions (Def), held by the testing authorities. Testing perennial ryegrass on 17 characters at 2 sites generated 48,960 observations and for tall fescue on 9 characters at 1 site, 12,960 observations (each for 3 accessions of 4 cultivars × 60 plants × 2 growing cycles). Distinctness required a p < 0.01 difference in a single character from the combined over years analysis (COYD). A few significant differences were recorded between E− and E+accessions. Cultivar Carn E+ was smaller than Carn E− for Infloresence Length (p < 0.01) in both years but COYD analysis (p < 0.05) was insufficient to declare distinctiveness. Overall, the number of observed differences between E−/E+ accessions was less or similar to the number expected purely by chance. In contrast, comparisons between Def and E− or E+ accessions showed a number of significant differences that were substantially more numerous than expected by chance. These results showed no conclusive evidence of endophyte inclusion creating false PBR distinctions but unexpectedly, several E− and E+ accessions were distinguished from their official definitive stock.

## Introduction

Vertically transmitting endophytes (*Epichloë* spp.) are non-sporulating asexually reproducing fungi of the *Clavicipitaceae* family, with no known soil borne resting spores^1^. They are indigenous in many soils and naturally occurring in many of the cool-season grass species of the Pooideae subfamily. Endophytes are present in approximately 20% of wild populations of European ryegrass and tall fescue and at higher levels in meadow fescue. The relatively low levels of endophyte occurrence is probably because the fungus-grass relationship is not a pure symbiotic one since the endophyte removes photosynthate resources from the grass and in times of shortage or stress can even deny the plant its requirements in a parasitic fashion^2^. However, evidence from New Zealand shows that selective increases in endophyte prevalence occurs in pastures where insect attack is a problem, but largely for those genotypes that confer an advantage to the host grass through the production of an insect toxin^3^.

Following the 2014 taxonomic revision of the genus Epichloë, the two species of endophyte that infect ryegrass are *E. festucae* var. lolii in perennial ryegrass (*Lolium perenne* L.) and *E. occultans* in Italian ryegrass (*Lolium multiflorum* Lam.*)*^4^. A single species (*E. coenophialum)*, infects tall fescue (*Festuca arundinacea* Schreb.), each as an intercellular fungus. There are four major groups of secondary metabolites that these fungi can produce: ergot alkaloids such as ergovaline and chanoclavine; indole diterpenes including lolitrem B and epoxy-janthitrem; pyrrolizidines which include lolines; the pyrrolopyrazine metabolite peramine^5–9^. Most strains of *E. festucae* var. *lolii* produce the alkaloid Lolitrem B^10^, which is a neurotoxin involved in the neuromuscular disorder ‘Ryegrass Staggers’^11^. In addition, is the metabolite Peramine, which deters feeding of insect adults and larvae at 10 ppm in artificial diets^12^. Likewise, in tall fescue, strains of the single infecting species, can produce the alkaloid ergovaline that causes ‘Fescue Toxicosis’ in livestock^13^. This toxin production capability is not an obligatory condition and strains that produce only one compound or none also exist, albeit these non-toxic strains are very rare in ryegrass and tall fescue.

As it has not been possible to induce a reproductive cycle *in vitro* it has not been possible to breed new endophyte variants. So all existing strains have been ‘discovered’ by screening existing populations and clonally multiplying selected isolates (GM and gene editing variants are now also possible but is beyond the scope of this study). Grass breeders have been able to find and incorporate endophyte strains that, for example, only carry the insect toxin and so do not impair the grazing stock and also ‘double zero’ strains that produce no toxins. It is for these ‘animal safe’ strains that plant breeders have more recently claimed agronomic advantages for farmers. This is due to these double-zero strains conferring greater environmental resilience and productivity to their host grass^14^.

There is evidence of endophyte presence conferring increased abiotic stress tolerance to the host plant. A 50% higher growth has been reported^15^ for endophyte infected plants at higher N levels, as well as increased tillering, plus greater tolerance and regrowth recovery from mild to severe moisture stress. An endophyte induced amelioration of drought stress has been shown in perennial ryegrass^16^ and recorded differences in tiller number, tiller length and shoot mass compared to endophyte free plants. Regarding the effect of endophyte presence on cultivar competitiveness^17^ found that endophyte carrying turf grass cultivars differed in measures of composition, structure and nutrient cycling from endophyte free cultivars. Recent work^18^ concluded that endophyte infection in perennial ryegrass could significantly increase days to heading and number of seeds per head, and decrease leaf length and the number of spikes per plant. This study further indicated that there were genetic differences between the cultivars. Although, it was unclear whether these differences were confounded with the level of endophyte infection, other work^19^ has found endophyte effects on grass cultivars to be variable.

Although not bred, endophyte strains are considered the property of the discoverer and in some cases patents have been taken out to protect this ownership. The equivalent protection system for the grass cultivars is the Plant Breeders Rights (PBR) statutory schemes (conforming to guidelines of the International Union for the Protection of New Varieties of Plants^20^) or as in some countries such as USA, similar ‘Plant Variety Protection’ schemes. This has introduced a potential for conflicting ownership and principles concerning how to describe and protect a new candidate grass cultivar that has been inoculated with an endophyte, whether patented or not. Furthermore, if the presence of endophyte can impact on a plant’s growth and morphology then it is possible that this could create a difference in the morphological plant characters used to assess the Distinctness, Uniformity and Stability (DUS) in PBR trials. The concern is that this could create an apparent distinctness between two accessions of the same cultivar, one with and the other without an endophyte inoculation. This would let an unscrupulous breeder circumvent the PBR protection on a competitor’s existing registered variety, by inoculating it with an endophyte. There is no consensus among testing authorities on how to manage this. Within the EU, submitting endophyte-carrying seed for testing is not permitted. However, New Zealand accepts such seed but requires full details of the endophyte strain and level of infection^21^.

The registration authorities could avoid any risk of plagiarism by requiring all seed submissions for PBR testing to be endophyte free. However, such a requirement would incur costly cleaning and seed stock management procedures, which the grass breeders’ representatives regard as unacceptable. Similarly, the breeders have concerns that they may incur seed certification problems when later seed lots containing endophyte were compared to definitive PBR stocks that would be endophyte free.

The current study compared example endophyte free, endophyte infected and official (endophyte free) ‘definitive’ seed stocks of cultivars of perennial ryegrass and tall fescue using official DUS spaced plant trials. The objective was to determine if the expression of the DUS characteristics of plants grown from endophyte infected seed lots was sufficiently modified to designate them as distinct from endophyte-free plants taken from the same seed lot of that cultivar or from its definitive stock.

## Materials and Methods

The plant material comprised of four cultivars of diploid amenity perennial ryegrass and four cultivars of hexaploid tall fescue. These were from different breeding programmes in the EU, NZ and USA and from known European/Australasia germplasm origins, to give a wide genetic and geographic base. All were EU registered cultivars and so previously tested and proven to be distinct, uniform and stable. For commercial in confidence reasons the registered names were replaced by codenames derived from Irish mountain names. The codenames were selected to have the same first letter as the original cultivar names as follows: Perennial ryegrass: Binnian, Carn, Croob and Gullion; Tall Fescue: Benbaun, Beann, Cove and Eagle.

The endophytes are defined as ‘wild type’ as they were not selectively screened to identify and isolate individual strain toxicity profiles. Hence all the ryegrasses were infected with the same *E. festucae* var. lolii inoculant producing Lolitrem-B and Peramine and the tall fescue with *E. coenophialum* producing Ergovaline and Loline.

Each cultivar was represented by two accessions, one containing endophyte (E+) and the other not containing endophyte (E−). These were produced by germinating out a large number of individual grass plants from each endophyte infected cultivar. As none of the seed lots had close to 100% of plants infected it was possible to screen for the presence or absence of endophyte and compile two sub-populations of plants from within each cultivar that were 100% endophyte infected and 100% endophyte free. This was done by agrinostic immunoblot SKU Number: Endo797-3 http://www.agrinostics.com/shop/. As per this previously published detection method^22^, the presence of *Epichloë* endophytes was assessed in two tiller sections from each plant, 6 weeks after transplanting, using the immunoblot assay according to the manufacturer’s description. The presence of endophyte was tested with an *in planta* assay using microsatellite markers B10 and B11^23^ on up to 200 tillers per accession. Tillers where no amplifications were detected were considered endophyte-free. From these resources, sixty plants were randomly selected to represent each E+ (100% endophyte) and E− (0% endophyte) accession. Accessions were tested and confirmed with the “Phytoscreen Field Tiller Endophyte Detection Kit”. In addition, sixty plants grown from the definitive seed sample of each cultivar, provided the third comparator accession (Def) in the experiment. These were the reference samples used annually in official DUS tests and were endophyte free as this is an existing condition for seed submitted to test authorities in the EU. Each centre provided plants from its own reference collection for these definitive (Def) accessions. In total 1440 plants (8 cultivars × 60 plants × 2 accessions E−/E+) were then established at the Examination Office (EO) test sites as part of the official the DUS trials.

The tall fescue accessions were planted at the official GEVES Examination Office (EO) site at L’Anjouère, France. For the perennial ryegrasses, each E− and E+ plant was split into two and grown on in multi-pots to provide two identical matching sets of plants. One set was planted into the official DUS trials at the Agri-Food and Biosciences Institute (AFBI), Crossnacreevy, Northern Ireland, UK and the other at the Federal Plant Variety Office (Bundessortenamt), Prüfstelle, Scharnhorst, Germany. This planting was repeated in the following year using new plants from the E−/E+ breeders’ resource and a new set of 60 seeds of each definitive accession, to give a full two-year DUS examination as routinely conducted by the EOs.

All test accessions were integrated into the regular DUS trials at each EO and examined using the Community Plant Varieties Office Technical Protocols^24^. This involved planting the 60 plants of each accession as individual spaced plants in the early summer and then recording on each plant the morphological characters listed in Tables 1 and 2. These recordings were conducted at the specified timings given in the CPVO protocol during the autumn of the year of sowing through to the summer of the following year. For the purpose of this paper shortened character names have been created for use in the data tables and text, as shown in Tables 1 and 2.

**Table 1:** Morphological characters examined in Perennial Ryegrass.

CPVO Code	Character Name (UK)	Shortened Character Name
2	Plant: vegetative growth habit (without vernalization)	Veg Habit − Vern.
4	Plant: width (after vernalization)	Width + Vern.
5	Growth of habit after vernalization	Habit + Vern.
6	Height of plant after vernalization	Height + Vern.
10	Plant: time of inflorescence emergence (after vernalization)	Time of Emerge
11	Plant: natural height at inflorescence emergence	Height @ Emerge
12	Plant: growth habit at inflorescence emergence	Habit @ Emerge
13	Flag leaf: length	Flag Length
14	Flag leaf: width	Flag Width
15	Flag leaf: length/width ratio	Flag L/W Ratio
16	Plant: length of longest stem, + inflorescence	Stem + Inflor. Length
17	Plant: length of upper internode	Internode Length
18	Inflorescence: length	Inflor. Length
19	Inflorescence: number of spikelets	Spiklet Number
20	Inflorescence: dens Inflorescence: density	Spiklet Density
21	Inflorescence: length of outer glume on basal spikelet	Glume Length
22	Inflorescence: length of basal spikelet excluding awn	Spiklet Length

CPVO -EU Community Plant Variety Office, Angers France – controller of PBR test schemes in EU.

**Table 2:** Morphological characters examined in Tall Fescue.

CPVO Code	Character Name (FR)	Shortened Character Name
6	Plant: natural height in autumn without vernalization	Height − Vern.
7	Plant: tendency to form inflorescences (without vernalization)	Inflor. − Vern
8	Plant: natural height after vernalization (4 weeks after beginning of vegetative growth)	Height + Vern.
9	Plant: time of inflorescence emergence (after vernalization)	Time of Emerge
10	Plant: growth habit at inflorescence emergence	Habit @ Emerge
11	Plant: natural height at inflorescence emergence	Height @ Emerge
12	Flag leaf: length	Flag Length
13	Flag leaf: width (same flag leaf as that used for 12)	Flag Width
14	Stem: length of longest stem including inflorescence (fully expanded)	Stem + Inflor. Length
15	Plant: length of upper internode (as for 14)	Internode Length
16	Inflorescence: length (as for 14)	Inflor. Length

CPVO -EU Community Plant Variety Office, Angers France – controller of PBR test schemes in EU.

The complete experimentation on perennial ryegrasses generated 48,960 observations, comprising 2 locations × 2 growing cycles × 4 varieties × 3 accessions × 60 plants/accession × maximum of 17 characters. Similarly, for tall fescue, a total of 12,960 observations were taken, comprising 1 location × 2 growing cycles × 4 varieties × 3 accessions × 60 plants/accession × maximum of 9 characters.

Statistical analyses were conducted according to the DUS testing guidelines of UPOV^20^ using the specifications for allogamous grasses. This involved the UPOV-approved statistical methods for determining the distinctness, uniformity and stability of candidate varieties, as used by all UPOV member state EOs. This is a Combined Over-Years Distinctness (COYD) analysis based on reported methods^25^. This incorporated a modified joint regression analysis (MJRA) model, which took account of systematic annual increases or decreases in character expression across all varieties by fitting extra terms, one for each year, in the analysis of variance. Each term represented the linear regression of the observations for the year against the cultivar means over both years^26^. This is an internationally recognised method which is incorporated into the protocols of UPOV^20^ and CPVO^24^, the full statistical methods for which are available in peer reviewed publication^25^^,^^26^. This is standard practice among workers in official plant registration schemes, for example across Europe, New Zealand and Canada.

As the objective of the study was to determine if the presence and absence of endophyte can create a PBR ‘distinction’ between accessions of the same cultivar, it is important to define the magnitude of difference required to designate them as ‘distinct’, independent varieties in these three official testing schemes. This is defined as the product of the COYD analysis tool and requires a combined over-two years difference at the 1% (P < 0.01) level in at least one character (assuming that the expression of that character is within a uniform range of expression and was not greater in one year and lesser in the other year, relative to the comparison accession).

## Results

The mean values for each character is presented for perennial ryegrass in Table 3 (Crossnacreevy) and Table 4 (Scharnhorst) and for tall fescue at L’Anjouère in Table 5. Each EO fully complied with the CPVO examination protocols for each species however for some characters alternative recording methods are permitted. Therefore, the Crossnacreevy data (Table 3) and the Scharnhorst data (Table 4) do not always report the observations in the same units. For example in Crossnacreevy, ‘Veg Habit –Vern’ is recorded as a scored estimate of the degree angle from the vertical and ‘Habit @ Emerge’ as the plant height:width ratio at ear emergence, while in Scharnhorst these two characters are recorded as notes on a 0–9 scale. As it was important to the objective of the study to comply with the DUS test procedures at each EO, the different recording values were retained so as to accurately emulate the normal tests employed at each EO. However, while Crossnacreevy records date of ear emergence as the number of days after the 1^st^ March and Scharnhorst records from the 1^st^ April, these data were standardised to 1^st^ March, to make direct comparisons of the data possible. Tall fescue ear emergence is recorded at L’Anjouère from 1^st^ January and was also standardized to 1^st^ March. Similarly, length units were standardised across all three EOs.

**Table 3:** Two year mean recordings of morphological characteristics of accessions of perennial ryegrass cultivars recorded at AFBI Crossnacreevy.

Shortened Character Name	Units	Binnian (def)	Binnian (E−)	Binnian (E+)	Carn (def)	Carn (E−)	Carn (E+)	Croob (def)	Croob (E−)	Croob (E+)	Gullion (def)	Gullion (E−)	Gullion (E+)
Veg Habit -Vern.	degree	45.18	44.88	44.88	43.38	44.53	44.92	40.04	41.44	41.28	44.00	46.00	46.14
Width +Vern.	cm	32.98	34.19	31.86	32.68	33.15	32.77	49.57	40.22	41.98	33.86	35.62	34.14
Habit +Vern.	degree	49.05	48.89	48.86	47.61	48.69	49.72	50.35	48.21	48.27	51.47	53.00	57.59
Height +Vern.	cm	25.98	25.55	25.17	23.39	24.77	25.44	37.85	31.08	31.49	29.52	30.83	32.60
Time of Emerge	*Day no	90.2	91.6	91.2	89.4	88.0	88.0	71.0	75.6	74.1	87.3	86.9	86.5
Height @ Emerge	cm	34.88	34.96	33.70	31.29	34.66	33.93	37.38	34.73	33.33	40.50	40.21	41.49
Habit @ Emerge	ratio	1.63	1.65	1.69	1.81	1.61	1.70	1.72	1.67	1.70	1.29	1.35	1.27
Flag Length	cm	13.80	14.64	14.30	14.15	14.53	14.84	15.02	14.10	14.04	15.29	16.34	15.66
Flag Width	mm	4.93	5.06	5.04	4.61	5.02	4.88	5.42	5.19	5.09	4.81	4.93	4.92
Flag L/W Ratio	ratio	2.93	3.20	2.89	3.16	3.02	3.23	2.85	2.86	2.82	3.30	3.40	3.40
Stem+Inflor. Length	cm	73.94	74.53	71.83	63.62	66.28	69.62	73.66	69.07	72.58	70.48	69.91	70.63
Internode Length	mm	27.32	28.04	26.40	23.85	24.54	24.95	20.20	20.88	20.77	23.56	22.36	21.94
Inflor. Length	cm	20.03	19.95	19.58	19.85	20.00	21.36	20.41	19.98	20.51	20.45	20.82	21.10
Spiklet Number	number	22.79	23.09	23.09	21.57	22.16	23.45	23.48	23.48	24.26	23.24	24.33	24.48
Spiklet Density	ratio	0.88	0.87	0.85	0.94	0.91	0.92	0.87	0.85	0.86	0.89	0.87	0.87
Glume Length	mm	8.36	8.34	8.30	8.91	8.62	9.22	9.18	8.98	8.87	8.95	9.00	8.83
Spiklet Length	mm	15.69	15.54	15.46	17.20	16.47	17.52	14.50	14.77	14.18	16.78	16.80	17.37

Def, definitive accessions; E−, endophyte free accessions; E+, endophyte inoculated accessions; * number of days after 1st March.

**Table 4:** Two year mean recordings of morphological characteristics of accessions of perennial ryegrass cultivars recorded at Bundessortentamt, Scharnhorst.

Shortened Character Name	Units	Binnian (def)	Binnian (E−)	Binnian (E+)	Carn (def)	Carn (E−)	Carn (E+)	Croob (def)	Croob (E−)	Croob (E+)	Gullion (def)	Gullion (E−)	Gullion (E+)
Veg Habit -Vern.	note	6.51	6.49	6.73	6.45	6.44	6.58	6.37	6.51	6.66	6.16	6.14	6.33
Width +Vern.	ratio	35.97	36.19	36.78	37.13	35.25	36.19	48.62	40.31	40.31	36.15	36.09	37.54
Time of Emerge	*Day no	87.6	86.9	86.9	85.6	83.9	84.8	66.8	68.2	69.3	81.3	81.7	82.0
Height @ Emerge	cm	45.28	45.95	46.17	45.69	44.92	45.53	46.70	42.72	42.31	46.94	46.48	47.26
Habit @ Emerge	note	5.37	5.26	5.27	5.12	5.14	5.22	4.99	4.9	4.88	5.12	5.02	5.02
Flag Length	cm	14.66	15.06	15.21	15.23	14.28	14.11	14.84	13.41	14.40	16.96	14.75	17.09
Flag Width	mm	3.86	3.93	3.77	3.60	3.72	3.66	4.48	4.13	4.37	3.71	3.8	4.01
Flag L/W Ratio	ratio	38.43	38.79	40.76	43.08	38.67	39.22	34.31	33.93	34.45	45.88	39.6	43.08
Stem+Inflor. Length	cm	64.14	63.03	61.77	60.58	59.7	60.01	65.26	63.42	62.08	58.01	60.35	59.64
Internode Length	mm	16.83	16.26	16.25	17.93	17.53	17.12	19.01	17.35	17.23	17.06	16.48	16.73
Inflor. Length	cm	22.07	20.86	21.31	19.01	20.28	20.33	17.43	19.8	19.39	16.22	18.99	17.55
Spiklet Number	number	19.50	19.18	19.24	19.64	19.59	19.07	18.22	18.6	18.44	18.99	19.17	18.82
Spiklet Density	ratio	86.52	84.96	84.64	91.38	89.85	89.93	95.96	94.44	94.18	89.81	86.17	88.9
Glume Length	mm	7.01	7.01	7.05	7.23	7.42	7.7	8.63	8.21	8.57	7.46	7.35	7.66
Spiklet Length	mm	11.06	10.6	10.66	10.81	11.81	11.54	12.48	12.22	12.7	11.46	11.91	11.33

Def, definitive accessions; E−, endophyte free accessions; E+, endophyte inoculated accessions; * number of days after 1st March.

**Table 5:** Two year mean recordings of morphological characteristics of accessions of tall fescue varieties recorded at GEVES, L’Anjouère.

Shortened Character Name	Units	Benbaun (def)	Benbaun (E−)	Benbaun (E+)	Beann (def)	Beann (E−)	Beann (E+)	Cove (def)	Cove (E−)	Cove (E+)	Eagle (def)	Eagle (E−)	Eagle (E+)
Height -Vern.	cm	19.08	20.30	21.13	14.50	13.10	15.75	13.50	14.93	16.29	14.32	17.08	17.12
Inflor. -Vern	1–9	2.47	2.60	2.60	6.57	4.54	5.18	6.78	4.91	5.19	5.46	4.25	3.97
Height +Vern.	cm	22.42	29.20	29.54	15.17	19.03	19.06	15.75	18.67	19.92	18.64	23.67	23.26
Time of Emerge	*day no	62.2	59.6	59.7	56.6	56.1	56.4	61.1	60.0	59.0	57.4	56.3	57.7
Habit @ Emerge	1–9	4.40	4.02	3.77	5.13	4.78	4.92	4.60	4.17	4.08	4.22	4.03	4.32
Height @ Emerge	cm	77.83	79.38	79.63	43.58	50.69	46.21	39.50	41.42	43.57	45.34	48.29	49.11
Flag Lf. Length	mm	175.78	201.01	200.12	123.33	116.56	126.71	97.22	103.13	105.69	120.14	125.08	123.36
Flag Lf. Width	mm	5.58	6.07	6.38	4.85	4.78	5.26	3.70	4.15	4.00	4.73	4.93	5.03
Stem+Inflor. Length	cm	161.42	161.89	161.67	116.92	119.23	119.04	94.08	96.90	97.15	112.29	112.83	114.07
Internode Length	mm	634.35	619.77	601.79	503.45	512.83	520.30	391.92	420.08	424.46	505.05	507.16	493.37
Inflor. Length	mm	257.05	260.59	266.21	185.42	184.48	188.64	139.17	144.74	151.49	177.82	181.89	173.27

Def, definitive accessions; E−, endophyte free accessions; E+, endophyte inoculated accessions; *number of days after 1st March.

As the expression of most of the measured characters are responsive to growing conditions there are notable differences in the magnitude of the results for the same cultivar at Crossnacreevy and Scharnhorst. So when the definitive stocks of the four perennial ryegrass varieties are compared between EOs, ear emergence was approximately 4.6 days later at Crossnacreevy than at Scharnhorst (approximately day 85 and 80.4 respectively). In making greatest to least comparisons across all twelve accessions, the differences in ear emergence was 20.6 days at Crossnacreevy and 20.8 days at Scharnhorst. On average these accessions had shorter vegetative plants (9.58 cm) and were wider (1.07 mm) and longer (0.9 cm) leaved than at Scharnhorst. The range in plant size was 17.7 cm for Width + Vern, 14.46 cm for Height + Vern and 10.20 cm for Height@Emerge at Crossnacreevy. At Scharnhorst the ranges were 13.37 cm and 4.54 cm for Width + Vern and Height@Emerge respectively. Likewise for leaf geometry Flag Length and Flag Width ranges were 2.54 cm and 0.81 mm at Crossnacreevy and 3.68 cm and 0.82 mm at Scharnhorst. Overall, however the definitive stocks of the four cultivars largely ranked in a similar order at each EO. As the tall fescue varieties were only examined at L’Anjouère a site to site comparison was not possible. However, as several characters are recorded at ear emergence then these records were being made between 1^st^ March and 24^th^ May in Crossnacreevy, 1^st^ March and 19^th^ May in Sharnhorst and 25^th^ April to 1^st^ May at L’Anjouère. The mean date of ear emergence (MDEE) or heading date of all cultivars and accessions (mean of Def, E−, and E+) at each EO is expressed as a mean date and as the range from the earliest to the latest heading cultivar. At Crossnacreevy the MDEE (day 85) had a range of 20.6 days; Scharnhorst MDEE (day 80.4) had a range of 21.2 days; and L’Anjouère MDEE (day 59) had a range of only 6 days. The ryegrass results reflect differing climatic conditions on the rate of physiological development, with Scharnhorst being earlier than Crossnacreevy but with a very similar range of heading dates for these identical plant accessions. The much narrower range across the tall fescue varieties indicates that MDEE was less discriminating than between the perennial ryegrasses.

The experimental design of four varieties by three accessions in each species, produced a total of 132 pair-wise comparisons (4 varieties × 3 accessions × 11 pair comparisons) at each EO, giving an overall total across all three test sites and both species of 396 pair comparisons. If these comparisons were presented for each examined character this would produce a total of 5,412 pair comparisons (132 pairs × 17 characters + 132 × 15 + 132 × 9). However, it is not necessary to provide tables of every comparison to understand the morphological responses to the E− and E+ treatments. Therefore, only a few examples are presented to represent how the pair-wise comparisons were made at each EO.

As defined in the Materials and Methods the minimum requirement for two accessions to be declared distinct is a p < 0.01 difference in at least one single character from the two-year COYD analysis. This level of distinction was met or exceeded in at least one character in all comparisons between the definitive stocks, confirming that these were four distinct ryegrass varieties and four distinct fescue varieties.

Significant differences were also recorded between E− and E+ accessions of the same cultivar. For example, in perennial ryegrass Carn E− and Carn E+ were found to be distinct at Crossnacreevy (Table 6) in two characters. Carn E+ was significantly smaller than Carn E− for Inflor. Length and Spikelet Length. For Inflor. Length this involved a p < 0.01 difference in each of the two test years but when analysed by COYD was found to only differ at p < 0.05, which is insufficient to designate these accessions as ‘distinct cultivars’. Similarly for Spikelet Length there was no significant difference in either test year but when these differences were combined over two years a p < 0.05 was achieved. It is notable that there were also significant differences in the first test year in Stem+Inflor. at p < 0.01 and Spikelet Length at p < 0.02 and in the second test year for Habit@Emerge at p < 0.05 and Spikelet Number at p < 0.01. However, none of these resulted in an over-years’ significance. Similarly, at Scharnhorst, Carn E− and Carn E+ were also found to differ over the two years at p < 0.05, but in this case in Width +Vern. (Table 7). In contrast the comparison between the E− and E+ accessions of the tall fescue cultivar Cove provided no significant differences either within individual years or in the combined over-years analysis, and so both versions of the cultivar were found to have remained indistinguishable and thus ‘not distinct’ in the DUS test requirement (Table 8).

**Table 6:** Example comparison of E− and E+ accessions of Carn perennial ryegrass by COYD analysis of two growing cycles at Crossnacreevy, N. Ireland.

CPVO Code	Shortened Character Name	2 yr Mean Difference	T Value	Probability		Difference	
						Year1	Year2
2	Veg Habit -Vern.	0.39	−0.46	64.277	ns	−	−
4	Width +Vern.	−0.38	0.24	81.385	ns	+	−
5	Habit +Vern.	1.03	−1.01	31.196	ns	−	+
6	Height +Vern.	0.67	−0.61	54.291	ns	−	+
10	Time of Emerge	0.08	−0.07	94.201	ns	+	−
11	Height @ Emerge	−0.73	−0.21	83.236	ns	−	+
12	Habit @ Emerge	0.09	−0.54	58.686	ns	+	−5
13	Flag Length	0.31	−0.56	57.587	ns	−	−
14	Flag Width	−0.14	0.89	37.254	ns	+	+
15	Flag L/W Ratio	0.21	−1.24	21.615	ns	−	−
16	Stem+Inflor. Length	3.34	−1.71	8.931	ns	−1	−
17	Internode Length	0.41	−0.49	62.178	ns	+	−
18	Inflor. Length	1.36	−2.38	1.814	*−	−1	−1
19	Spiklet Number	1.29	−1.66	9.801	ns	−	−1
20	Spiklet Density	0.01	−0.12	90.524	ns	−	+
21	Glume Length	0.60	−1.91	5.75	ns	−2	−
22	Spiklet Length	1.05	−2.00	4.652	*	−	−

Values positive if Carn E+ larger than Carn E−,+/−/ = if larger/smaller in each year-1 = p < 0.01, −2 = p < 0.02, −5 = p < 0.05, * = a distinction at p < 0.01 or greater.

**Table 7:** Example comparison of E− and E+ accessions of Carn perennial ryegrass by COYD analysis of two growing cycles at Scharnhorst, Germany.

CPVO Code	Shortened Character Name	2 yr Mean Difference	T Value	Probability		Difference	
						Year1	Year2
4	Width +Vern.	0.94	2.42	4.591	*	+5	
5	Habit +Vern.	0.14	1.01	34.763	ns	+	+
10	Time of Emerge	0.88	0.96	36.981	ns	+	+
11	Height @ Emerge	0.61	0.34	74.321	ns	+	+
12	Habit @ Emerge	0.08	0.7	50.855	ns	=	+
13	Flag Length	−1.64	−0.15	88.424	ns	-	+
14	Flag Width	−0.06	−0.29	78.179	ns	−	+
15	Flag L/W Ratio	0.55	0.37	72.634	ns	−	+
16	Stem+Inflor. Length	0.31	0.19	85.588	ns	−	+
18	Internode Length	−0.41	−0.62	55.976	ns	−	+
17	Inflor. Length	0.05	0.04	96.933	ns	−	−
19	Spiklet Number	−0.52	−2.42	5.192	ns	−	
20	Spiklet Density	0.09	0.05	96.000	ns	+	−
21	Glume Length	0.27	0.44	67.502	ns	−	+
22	Spiklet Length	−0.27	−0.22	83.294	ns	−	+

Values positive if Carn E+ larger than Carn E− +/−/= if larger/smaller/equal in each year, +5 = p < 0.05.

**Table 8:** Example comparison of E− and E+ accessions of Cove tall fescue as COYD analysis of two growing cycles at L’Anjouère, France.

CPVO Code	Shortened Character Name	2 yr Mean Difference	T Value	Probability		Difference	
						Year1	Year2
6	Height -Vern.	−1.35	−1.19	23.52	ns	−	−
7	Inflor. -Vern	0.862	0.7	0.388	ns	+	−
8	Height +Vern.	−1.25	−0.75	45.38	ns	−	+
9	Time of Emerge	0.96	−0.79	43.336	ns	+	−
10	Habit @ Emerge	−0.038	−0.04	59.14	ns	−	−
11	Height @ Emerge	−2.15	−0.82	41.128	ns	−	−
12	Flag Lf. Length	−2.59	−0.05	95.728	ns	−	+
13	Flag Lf. Width	0.15	0.78	43.743	ns	−	+
14	Stem+Inflor. Length	−0.27	−0.09	92.817	ns	+	−
15	Internode Length	−4.51	−0.22	82.962	ns	+	−
16	Inflor. Length	−6.7	−0.87	38.582	ns	−	−

Values positive if Cove E+ larger than Cove E−,+/− if larger/smaller in each year.

Tables 9 and 10 provide the overall summaries of significances for each ryegrass pair-wise comparison (Def/E−; Def/E+; E−/E+). This shows that only one E−/E+ comparison was DUS distinct (p < 0.01, Gullion at Scharnhorst). The other, below threshold (p < 0.05), significant differences were three of the four E−/E+ comparisons (Binnian, Croob and Gullion) at Crossnacreevy (Table 9) and one at Scharnhorst (Table 10, Carn). Among the four fescue varieties at L’Anjouère the only significant E−/E+ difference was for Beann E−/E+ at p < 0.05 (Table 11). In stark contrast, the vast majority of comparisons between the definitive stocks and either their E+ or E− version produced significant differences. At Crossnacreevy, Carn, Croob and Gullion were all significantly (p < 0.01 and p < 0.001) different and so above the DUS pass threshold, with only the Binnian E+ and E− accessions remaining indistinguishable from their definitive stocks. This was partially replicated at Scharnhorst as Carn E− and Gullion E− were distinct from their respective definitive stocks. At L’Anjouère, a high frequency of significant differences between the fescue definitive stocks and their E− or E+ equivalents was similarly observed, with Benbaun E− and E+, Beann E− and Cove E− all distinct from their definitive stocks at p < 0.01, with Cove E+ and Eagle E+ at p < 0.05. This meant that half of the fescue definitive versus E− or E+ comparisons were distinguished above the DUS threshold.

**Table 9:** Number of characters showing a significant difference in each accession in pair-wise comparisons for perennial ryegrass varieties at two sites after two growing cycles at Crossnacreevy, N. Ireland.

Accession	Definitive Accession (Def)				Endophyte Free Accession (E−)			
	P < 0.001	P < 0.01	P < 0.05	ns	P < 0.001	P < 0.01	P < 0.05	ns
Binnian (E−)	0	0	0	17	−	−	−	−
Binnian (E+)	0	0	0	17	0	0	1	16
Carn (E−)	0	3	3	11	−	−	−	−
Carn (E+)	0	2	0	15	0	0	2	15
Croob (E−)	3	0	2	12	−	−	−	−
Croob (E+)	3	0	1	13	0	0	0	17
Gullion (E−)	0	1	1	15	−	−	−	−
Gullion (E+)	0	1	0	16	0	0	1	16

Significances based on COYD analysis of two growing cycles, values show the number of characters expressing a difference at the denoted probability level. ns = number of characters showing no significant difference.

**Table 10:** Number of characters showing a significant difference in each accession in pair-wise comparisons for perennial ryegrass varieties at two sites after two growing cycles at Scharnhorst, Germany.

Accession	Definitive Accession (Def)				Endophyte Free Accession (E−)			
	P < 0.001	P < 0.01	P < 0.05	ns	P < 0.001	P < 0.01	P < 0.05	ns
Binnian (E−)	0	0	0	15	−	−	−	−
Binnian (E+)	0	0	0	15	0	0	0	15
Carn (E−)	0	0	1	14	−	−	−	−
Carn (E+)	0	0	0	15	0	0	1	14
Croob (E−)	0	0	0	15	−	−	−	−
Croob (E+)	0	0	0	15	0	0	0	15
Gullion (E−)	0	1	2	12	−	−	−	−
Gullion (E+)	0	0	0	15	0	1	0	14

Significances based on COYD analysis of two growing cycles, values show the number of characters expressing a difference at the denoted probability level. ns = number of characters showing no significant difference.

**Table 11:** Summary of significant difference occurrences in pair-wise comparisons between different accessions of the same tall fescue cultivar at L’Anjouère, France.

Accession	Definitive Accession (Def)				Endophyte Free Accession (E−)			
	P < 0.001	P < 0.01	P < 0.05	ns	P < 0.001	P < 0.01	P < 0.05	ns
Benbaun (E−)	1	1	3	5	—	—	—	—
Benbaun (E+)	1	3	1	4	0	0	0	9
Beann (E−)	1	2	3	3	—	—	—	—
Beann (E+)	0	0	0	9	0	0	1	8
Cove (E−)	1	0	3	5	—	—	—	—
Cove (E+)	0	0	1	8	0	0	0	9
Eagle (E−)	0	0	0	9	—	—	—	—
Eagle (E+)	0	0	2	7	0	0	0	9

Significances based on COYD analysis of two growing cycles, values show the number of characters expressing a difference at the denoted probability level. ns = number of characters showing no significant difference.

A further understanding of the nature of these differences can be derived from the number of characters that provided these differences at each probability level (Tables 9–11). For the E−/E+ comparisons, only Carn involved more than one character and only at p < 0.05 at Crossnacreevy, with the other five occurrences involving a single character. In stark contrast, there were up to six ryegrass characters showing significant differences in pair comparisons of definitive versus E− or E+ (Carn def to E−, 3 at P < 0.01 and 3 at P < 0.05). Equally notable were the definitive stock comparisons with Croob E− and with Croob E+, both of which recorded three character differences at p < 0.001. Among the fescue comparisons, differences from the definitive stocks were similarly strong, with occurrences in 4–6 of the nine characters measured in four of the eight Def/E− or Def/E+ comparisons.

In order to interpret these observations appropriately requires a consideration of the likelihood of these distinctions occurring purely by chance or whether they represent a cause and effect. For ryegrass, the total number of comparisons (character × accession pairs (Def/E− and Def/E+) × varieties) was 136 at Crossnacreevy and 120 at Sharnhorst and for the E−/E+comparisons were 68 and 60 respectively (Table 12). For fescue, the total number of comparisons were 72 (Def/E− and Def/E+) and 36 (E−/E+). It can be seen from Table 12, that the number of observed differences between E−/E+ in ryegrass was less or similar to the number expected purely by chance. The same outcome was found for the comparisons between the fescue E−/E+ accessions. For the comparisons between the definitive samples and the E− or E+ accessions, the number of observed differences was substantially greater than that expected by chance, in both ryegrass and fescue. Therefore, overall the results show no conclusive evidence of endophyte inclusion creating DUS distinctions, but that these test accessions (E−/E+) differed in a number of comparisons with their official definitive stock.

**Table 12:** Comparison of expected and observed significant differences between different accessions of the same cultivar.

	Comparison of E−,E+ and Def Accession			Comparison of E− and E+ Accession		
	P < 0.001	P < 0.01	P < 0.05	P < 0.001	P < 0.01	P < 0.05
**For perennial ryegrass at Crossnacreevy**						
	**17 Characters × 2 comparisons × 4 varieties = 136 pair contrasts**			**17 Characters × 1 comparison × 4 varieties = 68 pair contrasts**		
Expected	0.14	1.36	6.80	0.068	0.68	3.4
Observed	6	13	20	0	0	4
**For perennial ryegrass at Scharnhorst**						
	**15 Characters × 2 comparisons × 4 varieties = 120 pair contrasts**			**15 Characters × 1 comparison × 4 varieties = 60 pair contrasts**		
Expected	0.12	1.20	6.00	0.06	0.60	3.00
Observed	0	1	2	0	1	4
**For tall fescue at L’Anjouère**						
	**9 Characters × 2 comparisons × 4 varieties = 72 pair contrasts**			**9 Characters × 1 comparison × 4 varieties = 36 pair contrasts**		
Expected	0.07	0.70	3.50	0.04	0.36	1.80
Observed	4	10	23	0	0	1

## Discussion

The absence of distinguishing differences between E−/E+ accessions raises two initial questions. Firstly whether the E−/E+ accessions were correctly formulated. Given that the repeatability of the immunoblot assay used has been reported at around 97%^27^, incorrect assignment of tillers to E−/E+ accessions is a highly unlikely cause. Furthermore, by using the immunoblot approach it was possible to avoid any side effects of treating seed lots to remove endophyte, such as by heat treatment, that might impair germination or vigour or might incur a fitness sub-selection that could create apparent phenotypic divergences. Secondly it could be questioned whether it was reasonable to expect endophyte inoculation to cause any such effects. There is however, a considerable body of previously published studies that report notable differences between E− and E+ grasses. The symbiotic relationship between endophyte and grass is not strictly mutualistic as there can be negative implications for the host plant. For example, it has been found^28^ that some novel cultivar-endophyte associations could incur a yield disadvantage compared with endophyte free plants in the absence of insect herbivores. Similarly, infected tall fescue seed required more moisture to germinate and their seedlings more nutrients^29^, presumably due to the photosynthetic cost of supporting the fungus. In contrast there are also some reports of direct benefits of endophyte infection for the host plant. For example, an increased efficiency under low soil nitrogen for infected tall fescue has also been reported^28^, possibly through raised glutamine synthetase activity, while others have recorded enhancement of perennial ryegrass growth^30^. Furthermore, under mild to severe drought there is evidence of increased tillering and regrowth rates^15^, and changes in tiller number, tiller length and shoot mass^16^. Most dramatically of all, it has been discovered that endophyte infection triggered reprogramming of host metabolism^31^, favouring secondary metabolism at a cost to primary metabolism. It also induced changes in host development, particularly trichome formation and cell wall biogenesis. Therefore, it was reasonable to question whether inoculation with an endophyte might change a cultivars DUS identity.

The three EO sites represented a relatively wide geographic and climatic spread. Crossnacreevy (north of Ireland) is exposed to cool moist air from the maritime polar air mass. L’Anjouère (west coast of France) has a temperate maritime climate with a narrow annual temperature range. Scharnhorst (northern Germany) has very cold winters and rain spread evenly throughout the year. Despite these contrasting conditions there was no detectable evidence of climatic stress, such as drought, in either test year (nor of any insect attack). Therefore, any protective properties of the endophytes could not have benefited the grasses during the study. Furthermore, this null response is not in conflict with other published work. No significant differences were found in DM yield, DMD, WSC or CP between E− and E+ accessions of the perennial ryegrass cultivars AberDart and AberMagic^32^, while others also reported similar absence of responses in ryegrass and tall fescue^33,34^.

A further factor in fully understanding the implications of the current study is that infection responses appear to be more than endophyte strain specific, as detailed earlier, but can also depend on the genotype of the host plant. For example, host plant genotype specific differences in phenotypic responses have been reported^29,35^. While breeding for effective host-endophyte associations, changes in endophyte metabolite expression associated with host genotype and evidence of co-adaptation between plant and fungus have been reported^36^. This has been carried through to the commercial scale in fescues by selecting host genotypes that reduced the animal toxic ergovaline production of the endophyte^37^. Likewise, as the host genotype determines host growth and reproduction, this interacts with the endophyte biology^38^. Consequently, the findings of the current study cannot be deemed as a fundamental rule applicable to all grass-endophyte associations. Rather, it must be regarded to some extent as specific to these cultivar/strain symbionts. Nonetheless, the evidence does show that inoculating a registered cultivar with an endophyte that is novel to the host does not guarantee that the new association will be distinct from the original cultivar. This is more likely to be the case in DUS tests conducted under conditions where there are no detectable biotic or abiotic stress responses. In these circumstances, the endophyte cannot provide a resilience benefit to the host and so is less likely to induce any change in plant growth or productivity. This should allay, to some extent, the concern that an apparent PBR distinctness could occur between two accessions of the same cultivar, one with and the other without endophyte. However, to fully ensure that PBR protection can be retained for existing registered cultivars, further investigation of whether these findings can be repeated with a wider range of cultivar/strain symbionts, would be additionally informative. As would using test sites where biotic and abiotic stresses ranged from absent to prevalent.

The surprising result of the current study was the high frequency of significant differences and DUS distinctions between individual E− or E+ accessions and their equivalent definitive stocks. It is difficult to discount this as an aberration, given that all three EOs largely observed the same outcome. Each EO provided its own definitive stocks and so those from Crossnacreevy and Sharnhorst were not directly compared to confirm identicalness. However, they had been stored in ideal conditions from the time they were submitted for registration by the breeder. It is also a basic premise of the EU PBR scheme that definitive stocks at each EO are identical. This is to ensure the same protection exists across the entire EU, with validation tests and audit inspections conducted to impose this standardization. Therefore, this eliminates any concerns regarding the authenticity of the definitive stocks, particularly as any fault would have had to reoccur in several cultivars and two species at three sites, independently. The search for a cause therefore, switches to the E−/E+ samples provided by the breeders. The generation of the seed lots used in the trials was considered as a possible factor but as all lots were from certified seed stocks they were validated as complying with their definitive stocks. So this also seems an unlikely cause, particularly as any drift would have also had to occur in several cultivars and two species independently. The individual E−/E+ plants were produced by the breeders and posted to the EOs. This means that they had been treated differently to the definitive stocks, which were grown directly from seed. However, all plants were grown on in multi-pots before planting out. All centres reported successful establishment of all the measured plants, however, the German EO subsequently reported that the E−/E+ plants were much smaller when planted than those in the rest of the DUS trial, including their definitive stocks. While this might have affected development, particularly for the autumn recorded characters, it would be less expected to significantly affect growth the following year, when most characters were measured. Furthermore, as this difference did not occur at the other test centres, this does not provide a full or adequate explanation. The only other obvious step in the experimentation where accession differences could have been artificially created was during the compilation of the E− and E+ accessions. There is evidence that this was a risk to the study as the vertical transmission of endophytes has been shown to vary with host genotype^39,40^. Co adaptation of host and endophyte has also been reported^41^ and in breeding for successful grass/endophyte combinations^36^. This could mean that in subdividing each accession into E− and E+ plants, this had also divided the cultivar into two different types, one more conducive and one less conducive to endophyte transmission, with associated phenotypic differences. However, if this was the causal mechanism, then the E− and E+ accessions should have been distinct from each other, which they were not. So, at time of publication, the cause of the observed definitive stock differences remains unexplained.

## Conclusion

The potential for creating differences in a grass cultivar’s morphological identity by endophyte inoculation has implications for cultivar distinctiveness and for plant variety protection. The evidence from this study shows that inoculating a registered grass cultivar with an endophyte that is novel to the host does not ensure that the new association will be distinct from the original cultivar i.e. endophyte presence is not a reliable mechanism to change the DUS traits of a cultivar. This has importance for those bodies responsible for regulating and testing grass cultivars across Europe as well as the grass breeding industry. However, this cannot be interpreted as a universal rule for all grass-endophyte associations, rather the findings should be regarded as being new evidence, which at the very least, confirms that PBR distinguishing morphological changes are not an inevitable consequence of endophyte inoculation. It must also be stressed that in the three experimental sites there were no acute biotic or abiotic stresses, which reduced the opportunity for the endophyte to benefit the host grass in terms of growth and/or productivity. These implications must be taken into account when evaluating any other cultivar-endophyte associations and particularly when considering how to manage the DUS testing of cultivars that are commercialised as a cultivar/endophyte symbiont.
